# TRIM47: molecular characteristics, disease-related mechanisms, and clinical translational value

**DOI:** 10.3389/fimmu.2026.1830642

**Published:** 2026-05-28

**Authors:** Qingsong Wang, Xiaojian Zhuo, Xianmin Wang, Tongyong Luo, Jun Yin

**Affiliations:** 1 Department of Pediatrics, Public Relations Department, Ultrasound, West China Hospital Sichuan University Jintang Hospital/Jintang First People’s Hospital, Chengdu, Sichuan, China; 2 Pediatric Cardiology Center, Sichuan Provincial Women’s and Children’s Hospital/The Affiliated Women’s and Children’s Hospital of Chengdu Medical College, Chengdu, Sichuan, China

**Keywords:** E3 ubiquitin ligase, immunomodulation, nanoparticle, post-translational modification, targeted therapy, TRIM47, tumor microenvironment

## Abstract

TRIM47 is a core member of the tripartite motif (TRIM) family of E3 ubiquitin ligases, characterized by an N-terminal RBCC motif and a C-terminal PRY-SPRY domain that flexibly catalyze both K48- and K63-linked ubiquitination. Under physiological conditions, TRIM47 functions as a crucial safety valve for immunological and neurovascular homeostasis, primarily by degrading innate immune signaling hubs (such as MAVS) to prevent hematopoietic stem cell exhaustion and by stabilizing antioxidant responses in the central nervous system. However, in the tumor microenvironment (TME), TRIM47 undergoes profound functional reprogramming driven by specific post-translational modifications (PTMs) and altered substrate availability. This pathogenic shift redirects its ubiquitin ligase activity toward the targeted degradation of critical tumor suppressors and metabolic enzymes, while persistently activating pro-survival signaling cascades, such as the NF-κB and Wnt/β-catenin pathways. Consequently, TRIM47 drives malignant progression, metabolic reprogramming, and multidrug resistance across diverse anatomical systems, concurrently remodeling the TME toward an immunosuppressive state via altered metabolic byproducts. Clinically, elevated TRIM47 expression strongly correlates with advanced tumor stage, metastasis, and poor prognosis, establishing its robust potential as a pan-cancer predictive biomarker. Despite this, direct systemic therapeutic targeting of TRIM47 remains conceptually challenged by substantial on-target toxicities, including lethal autoimmune inflammation and blood-brain barrier disruption. This review systematically delineates the structural basis and context-dependent regulatory networks of TRIM47, critically evaluates its transition from a physiological guardian to a pathological driver, and assesses the translational feasibility and distinct obstacles of TRIM47-targeted precision nanomedicine.

## 1 Structural characteristics and physiological homeostasis of TRIM47

### 1.1 Structural architecture and substrate recognition

**Figure 1:**
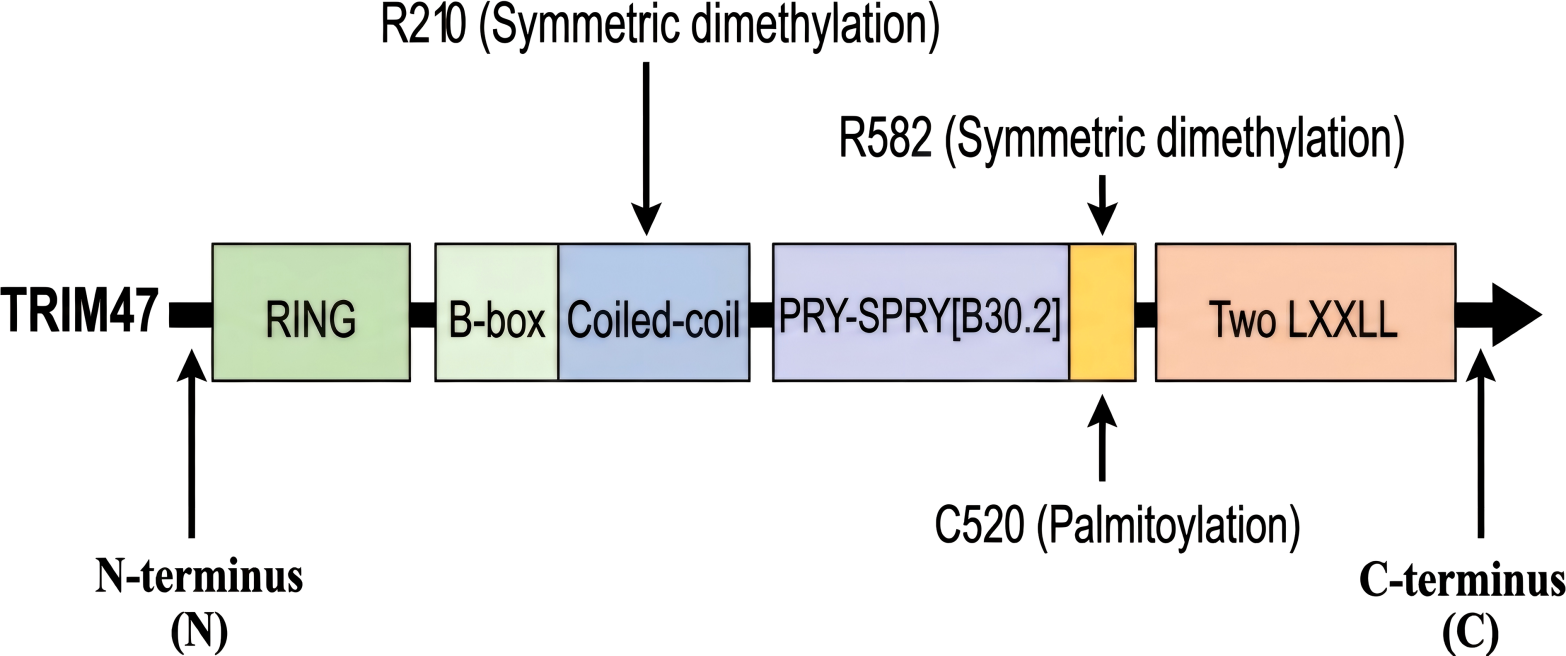
Schematic structure of human TRIM47 protein. The human TRIM47 protein comprises a conserved N-terminal tripartite motif—including a RING finger domain (conferring E3 ubiquitin ligase activity), a B-box domain, and a Coiled-coil domain—followed by a C-terminal PRY-SPRY domain responsible for substrate recognition. It harbors two LXXLL motifs facilitating nuclear receptor interaction. Crucial post-translational modifications, including symmetric dimethylation at R210/R582 and palmitoylation at C520, dictate its structural stability, subcellular localization, and downstream enzymatic activity.

The human TRIM47 gene, located on chromosome 17q25.1, encodes a core member of the tripartite motif (TRIM) family. The protein is composed of highly conserved functional domains that coordinately dictate ubiquitin catalysis, oligomerization, and signal transduction (1, 2). From the N-terminus to the C-terminus, the TRIM47 architecture consists of a RING finger domain, a B-box zinc finger domain, a coiled-coil domain, and a PRY-SPRY (B30.2) domain. The N-terminal RING domain serves as the primary catalytic core, engaging specific E2 ubiquitin-conjugating enzymes to initiate ubiquitin chain transfer. Experimental evidence indicates that mutations or deletions within this domain entirely abrogate its E3 ligase activity (3–5). The adjacent B-box and coiled-coil domains primarily mediate homo- or hetero-oligomerization, providing a structural scaffold that is essential for the assembly of stable cytoplasmic signaling complexes (6–9). The C-terminal PRY-SPRY domain functions as the decisive module for target substrate specificity. Recent molecular docking and immunoprecipitation studies have successfully mapped the exact interaction interfaces; for instance, the K534 and K600 residues of the TRIM47 PRY-SPRY domain directly engage the K342, W349, and E353 residues of hepatocyte nuclear factor 4 alpha (HNF4α) (10). Similarly, TRIM47 precisely targets the K51 residue of fructose-1,6-bisphosphatase 1 (FBP1) to initiate degradation (11). Furthermore, two unique LXXLL motifs located at the extreme C-terminus facilitate the binding of TRIM47 to various nuclear receptors, seamlessly integrating it into broader transcriptional regulatory networks (12–16) (**Figure 1**).

### 1.2 Post-translational modifications: the functional switches

**Figure 2:**
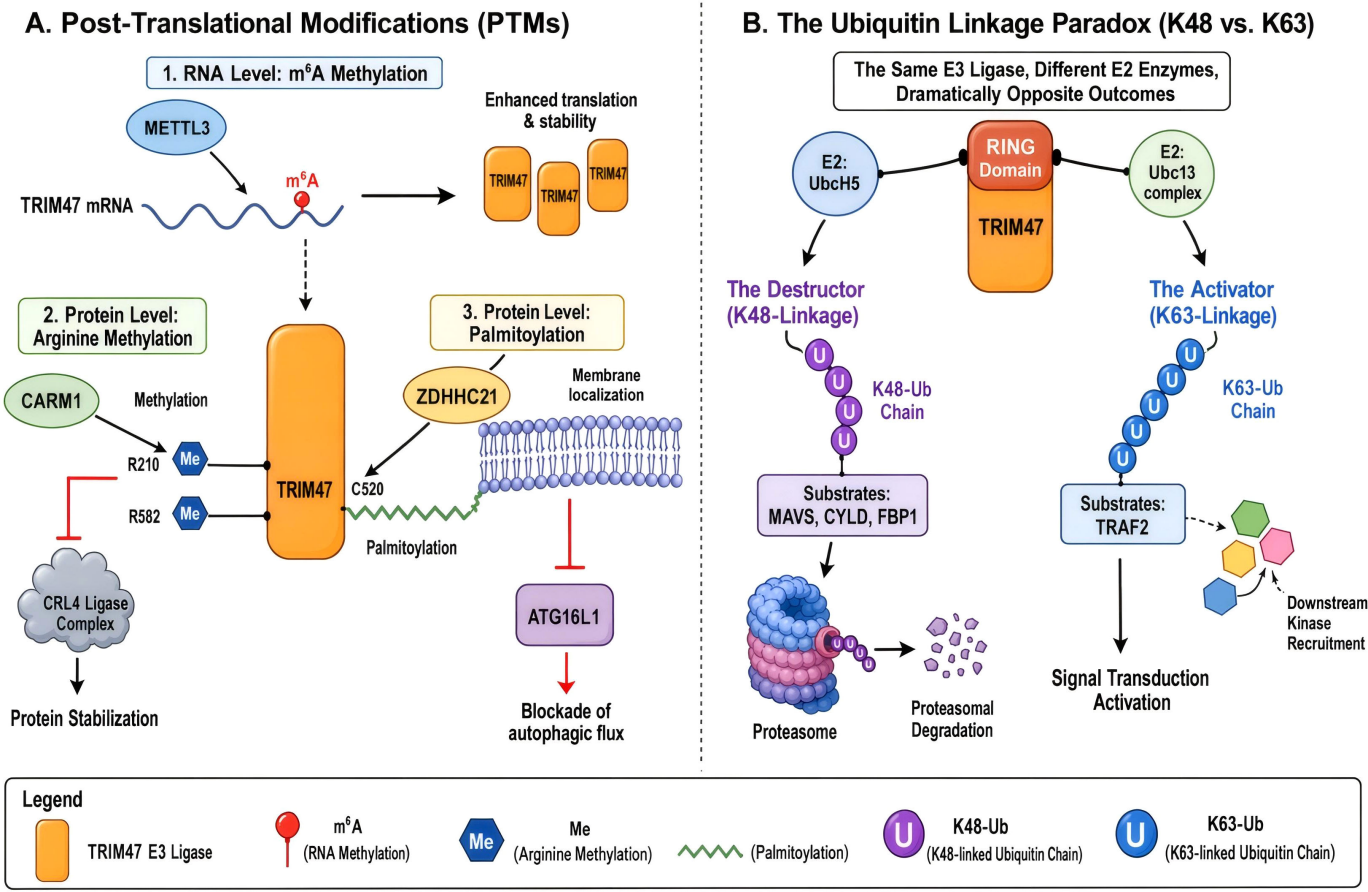
Regulatory mechanisms of TRIM47: Post-translational modifications (PTMs) and the ubiquitin linkage paradox. **(A)** PTMs as functional switches: Distinct modifications strictly dictate the fate of TRIM47. METTL3-mediated m6A methylation enhances its translation; CARM1-mediated arginine methylation shields it from proteasomal degradation; and ZDHHC21-mediated palmitoylation drives its membrane localization to block autophagy. **(B)** The ubiquitin linkage paradox: As a highly flexible E3 ligase, the catalytic outcome of TRIM47 depends on the specific E2 enzyme recruited. Binding with UbcH5 leads to K48-linked polyubiquitination for target degradation (e.g., MAVS, CYLD), whereas binding with the Ubc13 complex catalyzes K63-linked polyubiquitination, serving as a non-degradative scaffold for signal activation.

Beyond baseline transcriptional regulation, the intracellular stability, subcellular localization, and functional output of TRIM47 are dynamically governed by a sophisticated network of post-translational modifications (PTMs). These modifications serve as critical molecular switches that can lock TRIM47 into specific functional states, enabling rapid cellular adaptation to stress (17–20). For example, the protein arginine methyltransferase CARM1 dimethylates TRIM47 at residues R210 and R582. This arginine methylation effectively shields TRIM47 from recognition by the CRL4 ubiquitin ligase complex, thereby drastically prolonging its half-life and amplifying its downstream oncogenic signaling capacity (21–23). In parallel, lipid modifications also play a pivotal role. The palmitoyltransferase ZDHHC21 mediates the palmitoylation of TRIM47 specifically at the C520 site. This lipid attachment alters the membrane localization of TRIM47, facilitating its pathogenic interaction with targets such as ATG16L1 to block autophagic flux during acute inflammatory responses (24–26). Additionally, at the transcript level, the RNA methyltransferase METTL3 catalyzes the m^6^A methylation of TRIM47 mRNA, a modification that significantly enhances its transcript stability and subsequent protein translation efficiency (27, 28) (**Figure 2**).

### 1.3 TRIM47 in immunological and physiological homeostasis

Although initially characterized solely through its oncogenic properties, accumulating *in vivo* evidence has repositioned physiological TRIM47 as a fundamental maintainer of organismal homeostasis, functioning predominantly as an immunological brake and a neurovascular stabilizer. In the hematopoietic system, TRIM47 is constitutively expressed in hematopoietic stem cells (HSCs) and acts as an indispensable safety mechanism against stress-induced stem cell exhaustion. Under genotoxic stress or chronic inflammation, TRIM47 mediates the K48-linked ubiquitin-dependent degradation of the mitochondrial antiviral-signaling protein (MAVS). This degradation firmly suppresses the hyperactivation of the MAVS-type I interferon (IFN-I) innate immune axis, preventing the aberrant induction of cellular apoptosis (12, 29). This negative regulatory function conceptually parallels other immunosuppressive members of the TRIM family; for instance, TRIM26 similarly targets MAVS for degradation to blunt antiviral immunity, while TRIM25 and TRIM28 function as potent corepressors that upregulate PD-L1 and restrict the cGAS-STING pathways, respectively, collectively fostering an immunosuppressive microenvironment (30–36).Crucially, however, a gap remains in the current literature regarding the cell-autonomous expression and intrinsic function of TRIM47 in mature, terminally differentiated immune populations such as macrophages or T cells. Available data indicate that the immunomodulatory effects of TRIM47 on the broader immune microenvironment are predominantly secondary events, driven by the metabolic reprogramming of adjacent epithelial or tumor cells rather than direct immune cell regulation (37). Beyond the bone marrow, TRIM47 also finely tunes endothelial inflammation and neuroprotection. In vascular endothelial cells exposed to TNF-α stimulation, TRIM47 acts as a transient amplifier by mediating the K63-linked non-degradative ubiquitination of TRAF2, which rapidly upregulates adhesion molecules (ICAM-1, VCAM-1) for leukocyte recruitment and tissue repair (29).

TRIM47 exhibits specific CNS expression, localizing to presynaptic neuronal membranes and brain endothelial cells, with peak levels during the synaptic remodeling phase from embryonic stages to early adulthood (38). During rat hippocampal synaptogenesis, glutamate-induced, NMDA receptor-dependent activity dynamically upregulates TRIM47, which negatively regulates dendritic spine density and synaptic maturation; knockdown significantly increases excitatory synapse formation, indicating that TRIM47 acts as a physiological brake to prevent excessive synaptogenesis and maintain neural circuit precision (38). Beyond synaptic development, TRIM47 is indispensable for blood-brain barrier (BBB) integrity and neurovascular antioxidant defense. In brain microvascular endothelial cells, TRIM47 binds KEAP1, blocking KEAP1-mediated ubiquitination and degradation of NRF2 (39). This stabilizes NRF2 and activates antioxidant response elements (ARE), inducing heme oxygenase-1 (HO-1) and other cytoprotective enzymes. Trim47 knockout mice exhibit severe cognitive impairment, increased BBB permeability, and reactive astrogliosis, directly demonstrating the non-redundant role of TRIM47 in cerebrovascular homeostasis (39). TRIM47 gene variants also represent genetic risk factors for white matter hyperintensities (WMH). As an endogenous autophagy inhibitor highly expressed in brain endothelial cells, TRIM47 deficiency disrupts cerebral vascular structure, potentially mediating the pathological link between hypertension and age-related dementia (40). Furthermore, TRIM47 knockdown alleviates sevoflurane-induced neonatal hippocampal neuronal injury and cognitive deficits, concomitantly suppressing inflammatory responses and apoptosis, likely through downregulation of NF-κB signaling (41). Collectively, these findings establish TRIM47 as a pleiotropic guardian of nervous system homeostasis, operating through synaptic pruning, BBB maintenance, and antioxidant defense.

### 1.4 The ubiquitin linkage paradox: K48 vs. K63 selection and context-dependent regulatory logic

A defining biochemical characteristic of TRIM47 is its capacity to construct highly complex signaling networks by flexibly selecting between K48-linked ubiquitination, which directs substrates to the proteasome for degradation, and K63-linked ubiquitination, which serves as a non-degradative scaffold for signal transduction activation (4, 29, 42, 43). This dual-linkage catalysis is not a constitutive property but is precisely governed by three hierarchical layers of regulation, providing the molecular basis for TRIM47’s functional transition from physiological guardian to pathological driver.

#### 1.4.1 Substrate conformation and E2 enzyme coupling specificity

TRIM47 recognizes diverse substrates via its C-terminal PRY-SPRY domain, and the spatial microenvironment on the substrate surface determines which E2 ubiquitin-conjugating enzyme is recruited by the N-terminal RING domain (3). Conformations favoring the recruitment of E2s such as UbcH5 promote K48 chain assembly, routing target proteins for proteasomal degradation. In this context, TRIM47 mediates K48-linked ubiquitination and degradation of tumor suppressors and metabolic enzymes, including CYLD in gastric cancer (4), XAF1 in head and neck squamous cell carcinoma (44), and FBP1 in hepatocellular carcinoma, osteosarcoma, and pancreatic cancer (11, 15, 45). Conversely, conformations favoring the Ubc13 complex catalyze K63 chain formation, driving signal activation without substrate destruction. This mechanism is exemplified by TRIM47-mediated K63-linked ubiquitination of TRAF2 in vascular endothelial cells and NEMO in bronchial epithelial cells, leading to NF-κB and MAPK pathway activation (42, 43, 46).

#### 1.4.2 Post-translational modifications as molecular switches

The modification status of TRIM47 dynamically dictates its functional preferences and substrate spectrum. CARM1-mediated symmetric dimethylation at residues R210 and R582 shields TRIM47 from CRL4 recognition, prolonging its half-life and amplifying downstream oncogenic signaling capacity (9). This arginine methylation effectively locks TRIM47 into a pro-tumorigenic state, enhancing its capacity to degrade tumor suppressors such as HNF4α and SNAI1 (9, 10). In parallel, ZDHHC21-mediated palmitoylation at the C520 site alters the membrane localization of TRIM47, facilitating its pathogenic interaction with ATG16L1 to block autophagic flux during acute inflammatory responses (47). These PTMs do not merely modulate protein stability; they function as genuine molecular switches that redirect TRIM47 toward distinct substrate pools and ubiquitin chain linkages.

#### 1.4.3 Microenvironmental hijacking and target redirection

The flexibility in linkage selection explains the functional transition of TRIM47 between physiological and pathological states. Under normal physiological or stress conditions, TRIM47 serves as a critical safety valve for immune homeostasis, preferentially degrading MAVS via K48 chains to protect hematopoietic stem cells from inflammatory exhaustion (29). In parallel, it cooperates with other TRIM family members—such as TRIM25, which promotes pro-inflammatory signaling, and TRIM28, which suppresses antiviral immunity—to fine-tune innate immune responses (30, 31, 36). However, in the tumor microenvironment, this physiological mechanism is hijacked: the K48 ubiquitination capacity is redirected to massively clear tumor suppressors and metabolic enzymes (e.g., XAF1, FBP1, fumarate hydratase), while K63 ubiquitination persistently activates pro-survival pathways such as NF-κB (4, 42, 43). Notably, accumulation of metabolites resulting from metabolic enzyme degradation—such as fumarate from FH destruction—can further induce M2 macrophage polarization, remodeling an immunosuppressive microenvironment that fosters immune evasion (37).

In summary, the functional divergence of TRIM47 between physiological immune protection and pathological tumor promotion does not stem from alterations in the protein’s intrinsic enzymatic properties. Rather, it represents a functional reconfiguration of the same dual-linkage ubiquitination toolkit by varying substrate profiles, PTM status, and cellular microenvironment. This context-dependent regulatory mode—shaped by cell type, microenvironment, and stimulation conditions—provides a unified conceptual framework for understanding TRIM47-mediated pathogenesis and establishes its central role in immune homeostasis and disease transformation.

### 1.5 Commonalities and specificities between TRIM47 and other TRIM family members

TRIM47 embodies both the conserved structural hallmarks of the TRIM protein family and unique functional and disease-regulatory paradigms. Structurally, while all members share the core RBCC tripartite motif, TRIM47 strictly requires an intact RING domain for E3 ubiquitin ligase activity, with the C-terminal SPRY domain mediating substrate specificity. This distinguishes it from members such as TRIM29, which retain biological function despite lacking a canonical RING domain, and whose SPRY domain exhibits markedly different context-dependent functionality (3, 48). Mechanistically, consistent with most family members, TRIM47 regulates target proteins via ubiquitin-dependent pathways; however, it also executes non-ubiquitin-dependent functions (e.g., molecular scaffolding), reflecting contextual specificity not universally shared across the family (49). In disease biology, TRIM47 consistently presents a pro-oncogenic phenotype across human solid tumors, correlating with poor prognosis, metastasis, and drug resistance, whereas many other family members such as TRIM29 frequently exhibit “pro-oncogenic/tumor-suppressive” dual roles with greater heterogeneity (50). Regarding signal transduction, both TRIM47 and other family members engage core pathways including NF-κB and PI3K/AKT; however, TRIM47 uniquely targets critical regulatory nodes across multiple pathways in various cancers, highlighting its prominent hub characteristics. In non-oncological fields, research on TRIM47 has focused predominantly on hematopoietic/immune homeostasis and viral infections—a scope currently less extensive than in-depth studies of other members in pulmonary fibrosis and autoimmune diseases (51). In terms of therapeutic potential, due to its pan-cancer pro-oncogenic properties and association with resistance, TRIM47 offers broader clinical translational prospects compared to targets such as TRIM24, demonstrating substantial value in strategies aimed at overcoming multiple types of treatment resistance (43).

## 2 Pathological role of TRIM47 in human malignancies: a system-based perspective

**Figure 3:**
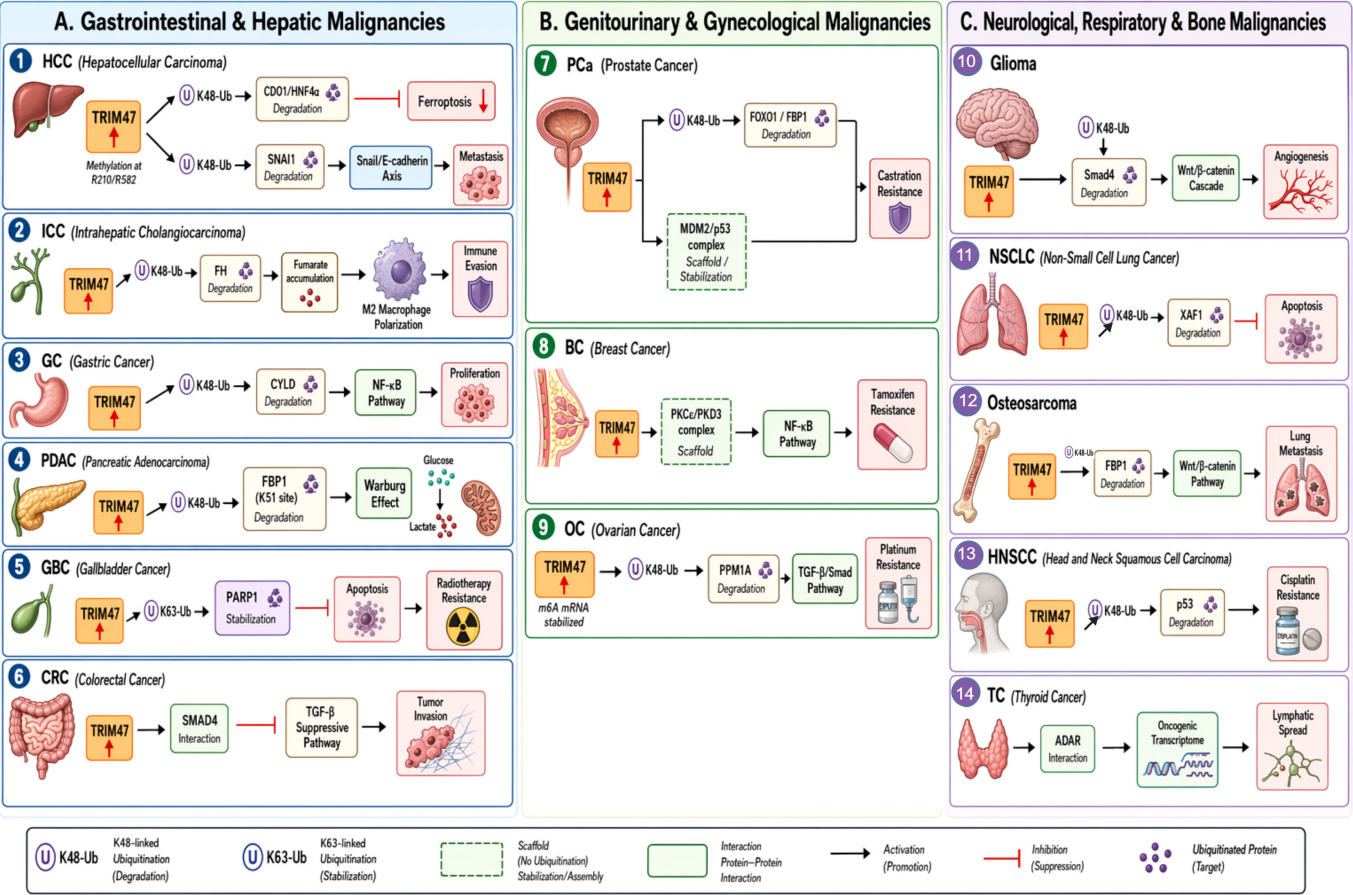
The context-dependent oncogenic roles of TRIM47 across diverse human malignancies. TRIM47 functions as a master facilitator of tumor progression and therapy resistance. **(A)** Gastrointestinal and Hepatic Malignancies: TRIM47 primarily drives metabolic rewiring (e.g., Warburg effect) and tumor microenvironment remodeling (e.g., macrophage polarization). **(B)** Genitourinary and Gynecological Malignancies: TRIM47 acts as a targeted survival shield, conferring profound resistance to endocrine and platinum-based therapies via ubiquitination or scaffolding functions. **(C)** Neurological, Respiratory & Bone Malignancies: TRIM47 promotes aggressive neoangiogenesis, lung metastasis, and suppresses cellular apoptosis via classical oncogenic cascades.

The aberrant upregulation of TRIM47 is a nearly ubiquitous phenomenon observed across a vast spectrum of human malignancies. Crucially, current transgenic mouse models lack evidence supporting TRIM47 as a spontaneous tumor initiator. Instead, the literature robustly defines TRIM47 as a highly potent master facilitator of malignant progression, metabolic reprogramming, and therapeutic resistance. By systematically categorizing its specific roles across different anatomical systems, a highly context-dependent network of TRIM47-mediated oncogenesis emerges(**Figure 3**).

### 2.1 Gastrointestinal and hepatic malignancies: integrating metabolism and immunity

In the digestive system, tumors are heavily influenced by chronic inflammation and metabolic stress. Here, TRIM47 serves as a crucial intracellular hub that orchestrates apoptosis resistance, drives metabolic shifts, and remodels the tumor microenvironment (TME).

Hepatocellular carcinoma (HCC) is characterized by insidious onset, high metastatic potential, and profound resistance to conventional therapies (52). Clinical data reveal that TRIM47 expression is significantly enriched in advanced HCC tissues (Stages III-IV) compared to early stages, correlating directly with poor patient survival (16). Mechanistically, TRIM47 protein is stabilized in HCC via CARM1-mediated arginine methylation at the R210 and R582 residues. This stabilized form actively promotes HCC metastasis by ubiquitinating and degrading the SNAI1 suppressor, thereby unleashing the Snail/E-cadherin epithelial-mesenchymal transition (EMT) axis (20, 21). Furthermore, TRIM47 acts as a central defender against lipid peroxidation by directly targeting CDO1 and HNF4α for degradation. The loss of these factors critically suppresses glutathione synthesis, thereby conferring profound ferroptosis resistance to HCC cells and allowing them to survive under severe oxidative stress (22, 23).

Similarly, intrahepatic cholangiocarcinoma (ICC) features a highly desmoplastic and immunosuppressive microenvironment (53). In this context, TRIM47 demonstrates its unique capacity to reshape local immunity through metabolic byproducts. TRIM47 directly ubiquitinates the metabolic enzyme fumarate hydratase (FH), leading to a massive intracellular accumulation of fumarate. When this altered metabolite is secreted into the TME, it acts as a potent oncometabolite that drives the polarization of adjacent tumor-associated macrophages (TAMs) toward an M2-immunosuppressive phenotype, effectively neutralizing local anti-tumor immune responses and facilitating immune evasion (25).

In the gastrointestinal tract, the progression of gastric cancer (GC) is heavily driven by chronic inflammation and is associated with a high frequency of regional lymphatic spread (54–56). Elevated TRIM47 levels in GC robustly correlate with larger tumor size and advanced TNM stage. At the molecular level, TRIM47 directly interacts with and degrades the cylindromatosis (CYLD) protein, a critical negative regulator of inflammation. The destruction of CYLD leads to the persistent hyperactivation of the NF-κB signaling pathway, sustaining malignant proliferation and inflammatory cell survival (57–59). Moreover, colorectal cancer (CRC) shares similar aggressive traits where metastasis remains the primary bottleneck in clinical management. In CRC tissues, overexpressed TRIM47 accelerates tumor invasion by directly engaging the SMAD4 pathway, thereby dampening the tumor-suppressive arm of TGF-β signaling and facilitating malignant spread (60).

In the accessory organs of digestion, pancreatic adenocarcinoma (PDAC) presents an exceptionally lethal clinical challenge due to its highly hypoxic and nutrient-deprived microenvironment (61, 62). To survive these harsh conditions, PDAC cells hijack TRIM47 for profound metabolic rewiring. Specifically, TRIM47 selectively targets the rate-limiting gluconeogenic enzyme FBP1 (binding precisely at the K51 residue) for K48-linked degradation. This targeted destruction relieves the metabolic suppression on aerobic glycolysis, thereby fueling the Warburg effect and ensuring rapid tumor growth despite nutrient scarcity (15). Concurrently, gallbladder cancer (GBC) is notorious for its extremely poor responses to clinical radiation therapy (63). In GBC, TRIM47 functions to limit genotoxic stress by directly degrading PARP1, which effectively suppresses DNA damage-induced apoptosis and confers substantial resistance to radiotherapy (64).

Across these gastrointestinal and hepatic malignancies, a distinct pattern emerges. Rather than merely acting as a canonical driver of cell cycle progression, TRIM47 consistently functions as a metabolic and inflammatory switch. By selectively degrading key enzymes like FBP1 and FH, its most profound pathological impact lies in utilizing altered metabolic networks to bridge intrinsic tumor survival with extracellular immune evasion.

### 2.2 Genitourinary, gynecological, and hormone-dependent malignancies: drivers of therapy resistance

In hormone-driven and genitourinary malignancies, the primary pathological role of TRIM47 prominently pivots toward maintaining cellular survival under severe therapeutic stress, particularly during endocrine deprivation and platinum-based chemotherapy. Prostate cancer (PCa), for instance, initially responds well to androgen deprivation therapy, but the transition to metastatic castration-resistant prostate cancer (mCRPC) represents a lethal clinical turning point with limited treatment options (65). TRIM47 expression is dramatically enriched in mCRPC tissues compared to hormone-sensitive early stages. Functionally, it facilitates castration resistance through multiple converging molecular axes. It directly ubiquitinates the tumor suppressor FOXO1 for degradation, selectively degrades FBP1 to shift metabolic dependency, and stabilizes the MDM2/p53 complex. These actions collaboratively drive unchecked cell cycle progression and tumor survival despite the absolute blockade of androgen signaling (66, 67). Similarly, in estrogen receptor-positive (ER+) breast cancer, the acquisition of resistance to endocrine therapies remains a major obstacle (68, 69). Elevated expression of TRIM47 in breast cancer tissues serves as a strong predictive marker for poor responses to Tamoxifen. At the molecular level, TRIM47 mediates the ubiquitination and degradation of PKC-ϵ, a process that paradoxically activates downstream survival cascades, allowing breast cancer cells to bypass Tamoxifen-induced growth arrest and continue malignant proliferation (8, 13, 18).

In gynecological oncology, ovarian cancer (OC) is notoriously characterized by late-stage diagnosis and the rapid acquisition of severe chemoresistance (70–72). In OC cells, high levels of TRIM47 mRNA are structurally sustained by METTL3-mediated m6A modification, ensuring robust protein translation (73). The resulting overexpressed TRIM47 protein selectively targets and degrades PPM1A. The removal of PPM1A effectively releases the inhibitory brake on the TGF-β/Smad signaling pathway, an activation that aggressively promotes epithelial-mesenchymal transition (EMT) and significantly blunts the cytotoxic effects of standard Platinum-Paclitaxel chemotherapy regimens (7). Collectively, in these hormone-driven and genitourinary malignancies, TRIM47 operates fundamentally as a biological survival shield. Its targeted degradation of specific intracellular enzymes uniformly converges on a single, devastating clinical phenotype: the circumvention of standard-of-care treatments, firmly establishing TRIM47 as an ideal predictive biomarker for therapeutic failure.

### 2.3 Neurological, respiratory, and bone malignancies

In highly heterogeneous and aggressive solid tumors spanning other anatomical systems, TRIM47 frequently acts as an indispensable driver of neoangiogenesis and an ultimate suppressor of cellular apoptosis. Glioma is the most common and lethal primary malignant brain tumor in adults, notoriously characterized by rapid invasive growth, robust pathological angiogenesis, and a dismal prognosis due to the near impossibility of complete surgical resection (74–78). In glioma tissues, elevated TRIM47 levels tightly correlate with higher WHO grading, increased microvessel density, and shortened overall patient survival. Mechanistically, TRIM47 directly ubiquitinates the tumor suppressor Smad4, which relieves structural transcriptional repression and subsequently hyperactivates the oncogenic Wnt/β-catenin signaling cascade (79, 80). This axis aggressively promotes both the uncontrolled proliferation of glioma cells and the pathological formation of the tumor vasculature network (81).

In the respiratory system, lung cancer remains the leading cause of cancer-related mortality worldwide, with non-small cell lung cancer (NSCLC) accounting for the vast majority of cases (81, 82). The acquisition of resistance to cellular apoptosis is a major hurdle in NSCLC targeted therapies (83). Overexpressed TRIM47 promotes NSCLC malignant progression by inactivating p53-mediated cell cycle surveillance and concurrently activating NF-κB-dependent epithelial-mesenchymal transition (EMT) signaling, thereby facilitating unrestricted proliferation and metastatic dissemination (84).Similarly, osteosarcoma (OS) is the most frequent primary malignant bone tumor affecting children and adolescents, with rapid pulmonary metastasis acting as the primary cause of specific lethality (85–87). TRIM47 acts as a critical promoter of this metastatic spread. Similar to its metabolic role in pancreatic cancer, TRIM47 targets and degrades the metabolic enzyme FBP1 in OS cells. This targeted degradation subsequently upregulates the Wnt/β-catenin pathway, significantly accelerating the rate of lung metastasis *in vivo* (29). Furthermore, In head and neck squamous cell carcinoma (HNSCC), TRIM47 degrades XAF1 (XIAP-associated factor 1), attenuating XAF1-mediated apoptotic and autophagic pathways to promote cancer cell survival and cisplatin resistance (44, 88–91).In thyroid cancer (TC), its functional interaction with ADAR regulates local oncogenic transcriptomes, a process that strongly correlates with advanced lymphatic spread and poor clinical outcomes (48–50) (**Table 1**).

**Table 1:** TRIM47 in diseases: expression, mechanisms, and quantitative evidence.

Disease category	Specific disease	TRIM47 expression	Core mechanisms in your manuscript	References
Digestive System Tumors	Hepatocellular Carcinoma (HCC)	Upregulated in advanced stages	Promotes metastasis via SNAI1; induces ferroptosis resistance by degrading CDO1/HNF4α	(9, 10, 16)
	Intrahepatic Cholangiocarcinoma (ICC)	Significantly upregulated	Degrades FH, increases fumarate, induces M2 macrophage polarization	(38, 92)
	Gastric Cancer (GC)	Upregulated with TNM stage	Ubiquitinates and degrades CYLD, activates NF-κB	(4, 57, 58)
	Gallbladder Cancer (GBC)	Highly expressed	Stabilizes PARP1 to promote DNA repair and resistance to radiotherapy	(64)
	Pancreatic Ductal Adenocarcinoma (PDAC)	Highly expressed	Degrades FBP1 to enhance aerobic glycolysis (Warburg effect)	(15)
Genitourinary & Gynecologic Tumors	Prostate Cancer (PCa)	Higher in mCRPC	Degrades FOXO1 and FBP1; regulates MDM2/p53 pathway	(66, 67)
	Breast Cancer (BC)	High in ER+ tamoxifen-resistant tumors	Stabilizes PKCϵ/PKD3 to activate NF-κB and induce endocrine resistance	(13, 18)
	Ovarian Cancer (OC)	Highly expressed in chemoresistant tumors	Degrades PPM1A, activates TGF-β pathway, promotes EMT and chemoresistance	(7)
Other Malignancies	Glioma	Increased with WHO grade	Ubiquitinates Smad4, activates Wnt/β-catenin, promotes angiogenesis	(79–81)
	Non-Small Cell Lung Cancer (NSCLC)	Upregulated	Inhibits p53; activates NF-κB-EMT pathway	(84)
	Head and Neck Squamous Cell Carcinoma (HNSCC)	Highly expressed	Degrades XAF1 to promote survival and cisplatin resistance	(44)
	Osteosarcoma (OS)	Highly expressed	Degrades FBP1, activates Wnt/β-catenin, promotes lung metastasis	(45)
	Thyroid Cancer (TC)	Higher in ATC	Ubiquitinates ADAR to promote progression and lymphatic metastasis	(50)
Non-Neoplastic Diseases	Cerebral Small Vessel Disease (cSVD)	Endothelium-specific	Stabilizes NRF2 via KEAP1 interaction to maintain blood–brain barrier	(39, 40)
	Sepsis/Acute Lung Injury (ALI)	Upregulated in lung endothelium	Palmitoylation-dependent; inhibits ATG16L1 and autophagy; aggravates inflammation	(46, 47)
	Asthma	Upregulated in airway epithelium	Promotes NF-κB/NLRP3 activation and epithelial pyroptosis	(43)

### 2.4 Shared oncogenic mechanisms and therapeutic resistance patterns across tumor types

Whilst the preceding system-based categorization highlights the context-specific pathological roles of TRIM47, a striking convergence of core mechanisms emerges across diverse malignancies. This pan-cancer functional homology underscores the therapeutic rationale for targeting TRIM47 as a universal master facilitator of malignant progression.

#### 2.4.1 Metabolic reprogramming as a universal axis

Across gastrointestinal, genitourinary, and bone malignancies, TRIM47 consistently targets rate-limiting metabolic enzymes for K48-linked degradation, driving the Warburg effect and conferring ferroptosis resistance. In hepatocellular carcinoma, pancreatic adenocarcinoma, and osteosarcoma, degradation of fructose-1,6-bisphosphatase 1 (FBP1) relieves gluconeogenic suppression, accelerates aerobic glycolysis, and sustains rapid tumor growth under nutrient deprivation (11, 15, 29). Concurrently, in hepatocellular carcinoma, TRIM47-mediated destruction of cysteine dioxygenase 1 (CDO1) and hepatocyte nuclear factor 4 alpha (HNF4α) suppresses glutathione synthesis, conferring profound resistance to lipid peroxidation and ferroptosis (10, 16). In intrahepatic cholangiocarcinoma, a distinct but convergent metabolic mechanism operates: TRIM47 ubiquitinates fumarate hydratase (FH), causing intracellular fumarate accumulation that is secreted into the tumor microenvironment to drive M2 macrophage polarization and establish an immunosuppressive milieu (37). These findings establish metabolic enzyme degradation as a recurring oncogenic strategy that bridges intrinsic tumor survival with extracellular immune evasion.

#### 2.4.2 Persistent activation of pro-survival signaling cascades

Independent of anatomical origin, TRIM47-mediated degradation of negative regulators sustains hyperactivation of NF-κB, Wnt/β-catenin, and TGF-β signaling. In gastric cancer, destruction of the deubiquitinase CYLD unleashes NF-κB-driven proliferation and inflammatory survival (4); in breast cancer, stabilization of the PKCϵ/PKD3 complex via scaffolding function sustains NF-κB activation and drives endocrine therapy resistance (13); in glioma and osteosarcoma, degradation of Smad4 and FBP1, respectively, converges on Wnt/β-catenin hyperactivation to promote angiogenesis and metastasis (80, 81). This signaling convergence suggests that TRIM47 functions not merely as a pathway-specific modulator, but as a multi-hub amplifier that integrates oncogenic inputs across disparate cellular contexts.

#### 2.4.3 Therapeutic resistance: four convergent modes

Analysis across tumor types reveals four recurring resistance modalities orchestrated by TRIM47 (**Table 2**). First, enhanced DNA damage repair through K63-linked stabilization of PARP1 in gallbladder cancer accelerates double-strand break repair and confers radioresistance (64). Second, metabolic reprogramming via FBP1 or FH degradation constructs an immunosuppressive microenvironment that hinders immune checkpoint blockade efficacy (11, 15, 37). Third, hormone and signal bypass activation—exemplified by MDM2/p53 complex stabilization in prostate cancer and PKCϵ/PKD3 scaffolding in breast cancer—allows tumor cells to circumvent androgen or estrogen deprivation (13, 66). Fourth, degradation of pro-apoptotic proteins such as XAF1 in head and neck squamous cell carcinoma and PPM1A in ovarian cancer establishes a survival defense that blunts chemotherapy-induced programmed cell death (7, 29).

**Table 2:** TRIM47-mediated therapy resistance.

Resistance mode	Core mechanism	Key substrates/pathways	Typical therapy	Typical tumors	References
Enhanced DNA damage repair	Stabilizes DNA repair proteins to evade apoptosis	PARP1, NF-κB	Radiotherapy; Platinum chemotherapy	Gallbladder cancer	(64)
Metabolic reprogramming	Degrades metabolic enzymes to drive Warburg effect and immune suppression	FBP1, FH, HNF4α	Chemotherapy	HCC, ICC, PDAC, Osteosarcoma	(10, 15, 38, 45)
Hormone/signaling bypass	Stabilizes receptors or activates bypass signaling	AR/ER, PKCϵ/PKD3, SMAD4	Endocrine therapy; Targeted therapy	Breast cancer; Prostate cancer; NSCLC	(13, 66)
Apoptosis/autophagy inhibition	Degrades pro-apoptotic proteins to promote survival	XAF1, PPM1A	Chemotherapy	HNSCC; Ovarian cancer	(7, 44)

#### 2.4.4 Immunomodulatory convergence: the “cold tumor” phenotype

A critical shared consequence of TRIM47 activity across multiple solid tumors is the remodeling of the tumor microenvironment toward an immunosuppressive state. In intrahepatic cholangiocarcinoma, fumarate-driven M2 macrophage polarization actively recruits regulatory T cells and excludes cytotoxic CD8+ T cell infiltration, creating an “immune desert” phenotype (37). In gastric cancer, NF-κB hyperactivation upregulates pro-inflammatory cytokines that sustain chronic inflammation while paradoxically fostering immune tolerance (4). Although direct ubiquitin modification of immune checkpoint molecules such as PD-L1 by TRIM47 has not been experimentally validated, the indirect establishment of an M2-type, PD-L1–enriched suppressive microenvironment provides a mechanistic rationale for combining TRIM47 inhibition with immune checkpoint blockade (37). These convergent immunomodulatory effects position TRIM47 as a pan-cancer determinant of immunotherapy response.

## 3 TRIM47 in non-neoplastic diseases: the dual-faced regulator of homeostasis

While TRIM47 undoubtedly acts as an aggressive oncogenic driver within the tumor microenvironment, its physiological expression in normal tissues is paradoxically essential for maintaining hematopoietic, inflammatory, and neurovascular homeostasis. This contextual duality highlights the extreme complexity of systemic targeted interventions.

### 3.1 Hematopoietic stress and lethal cytokine storms

**Figure 4:**
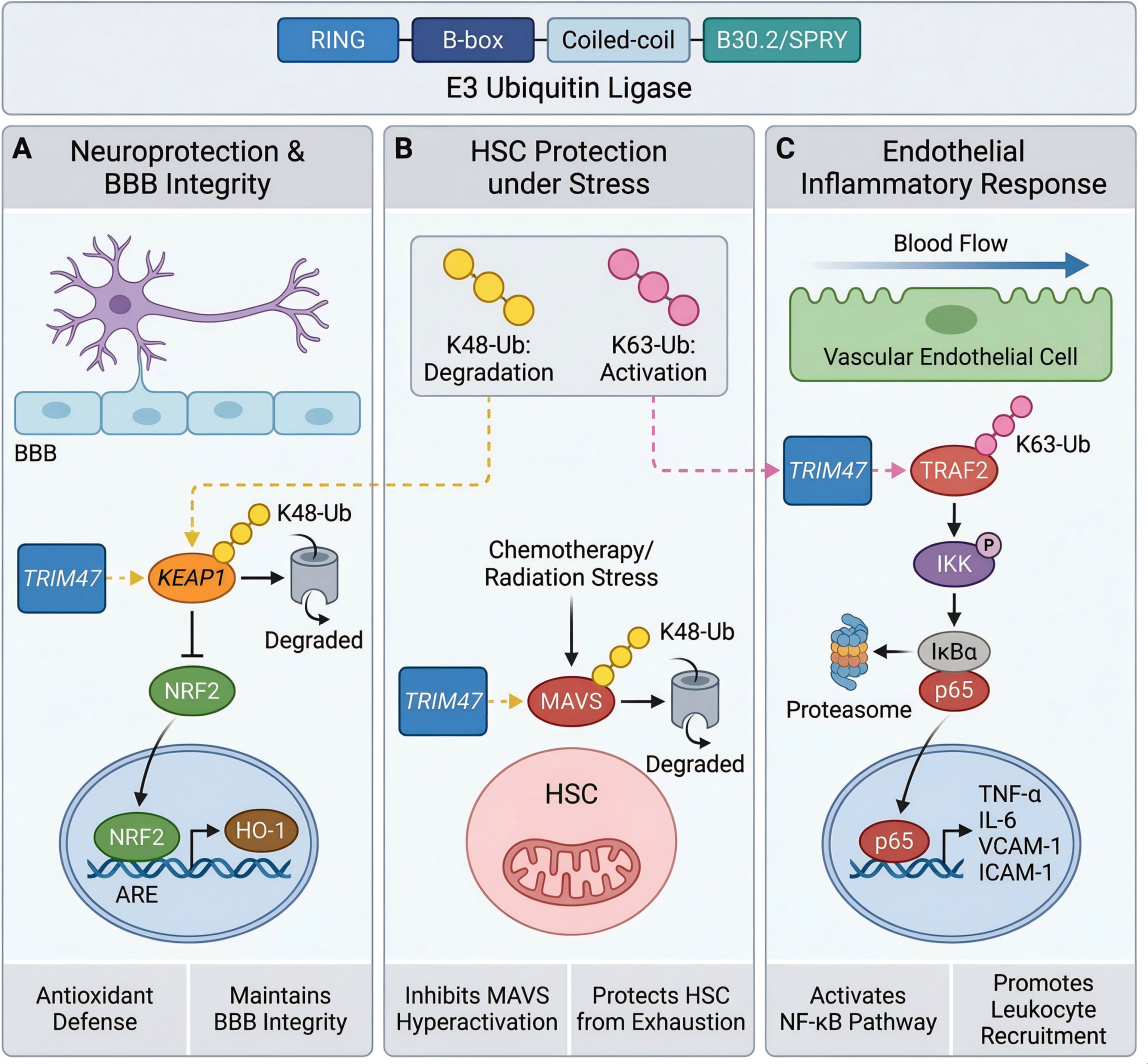
Context-dependent functions of TRIM47. TRIM47, an E3 ubiquitin ligase, exerts distinct regulatory roles depending on cellular context. **(A)** In neurons, TRIM47 facilitates K48-linked ubiquitination and degradation of KEAP1, thereby releasing NRF2 to activate antioxidant response elements (ARE) and induce HO-1 expression, promoting neuroprotection and maintaining blood-brain barrier (BBB) integrity. **(B)** Under chemotherapy or radiation stress, TRIM47 mediates K48-linked ubiquitination and degradation of MAVS in hematopoietic stem cells (HSCs), preventing excessive innate immune activation and protecting HSCs from exhaustion. **(C)** In endothelial cells, TRIM47 promotes K63-linked ubiquitination of TRAF2, activating the IKK-IκBα-p65 axis to induce NF-κB signaling, resulting in the upregulation of pro-inflammatory cytokines (TNF-α, IL-6) and adhesion molecules (VCAM-1, ICAM-1), thereby driving leukocyte recruitment.

In clinical oncology, systemic chemotherapy and total body irradiation frequently induce severe bone marrow suppression, representing a life-threatening clinical challenge. The maintenance and survival of hematopoietic stem cell (HSC) pools during such extreme physiological stress rely on the precise control of innate immune pathways. A landmark recent study has robustly redefined physiological TRIM47 as the master safety valve guarding HSCs against stress-induced exhaustion (29). Under steady-state conditions, Trim47 knockout (Trim47^−^/^−^) mice exhibit normal physiological development without spontaneous abnormalities. However, upon exposure to severe genotoxic stress, such as 5-FU chemotherapy or systemic irradiation, the absence of TRIM47 triggers catastrophic biological consequences. Mechanistically, physiological TRIM47 limits hyperactive innate immunity by mediating the K48-linked ubiquitination and degradation of MAVS. When TRIM47 is depleted, MAVS abnormally and massively accumulates, leading to persistent hyperactivation of the downstream TBK1/IRF3 and NF-κB inflammatory pathways. This signaling anomaly initiates a deadly systemic cytokine storm driven primarily by explosive cellular secretions of interleukin-6 (IL-6), tumor necrosis factor-alpha (TNF-α), and interleukin-1 beta (IL-1β), which collectively induce severe and irreversible multi-organ damage (29). Concurrently, the massive pathological overproduction of Type I interferons (IFN-α and IFN-β) directly inhibits HSC self-renewal capacity and actively drives the stem cell pool into apoptosis. Consequently, stressed Trim47^−^/^−^ mice rapidly succumb to lethal systemic inflammation and complete hematopoietic failure, underscoring the indispensable nature of TRIM47 in steady-state immune survival (**Figure 4**).

### 3.2 Neurovascular integrity and pulmonary inflammation

**Figure 5:**
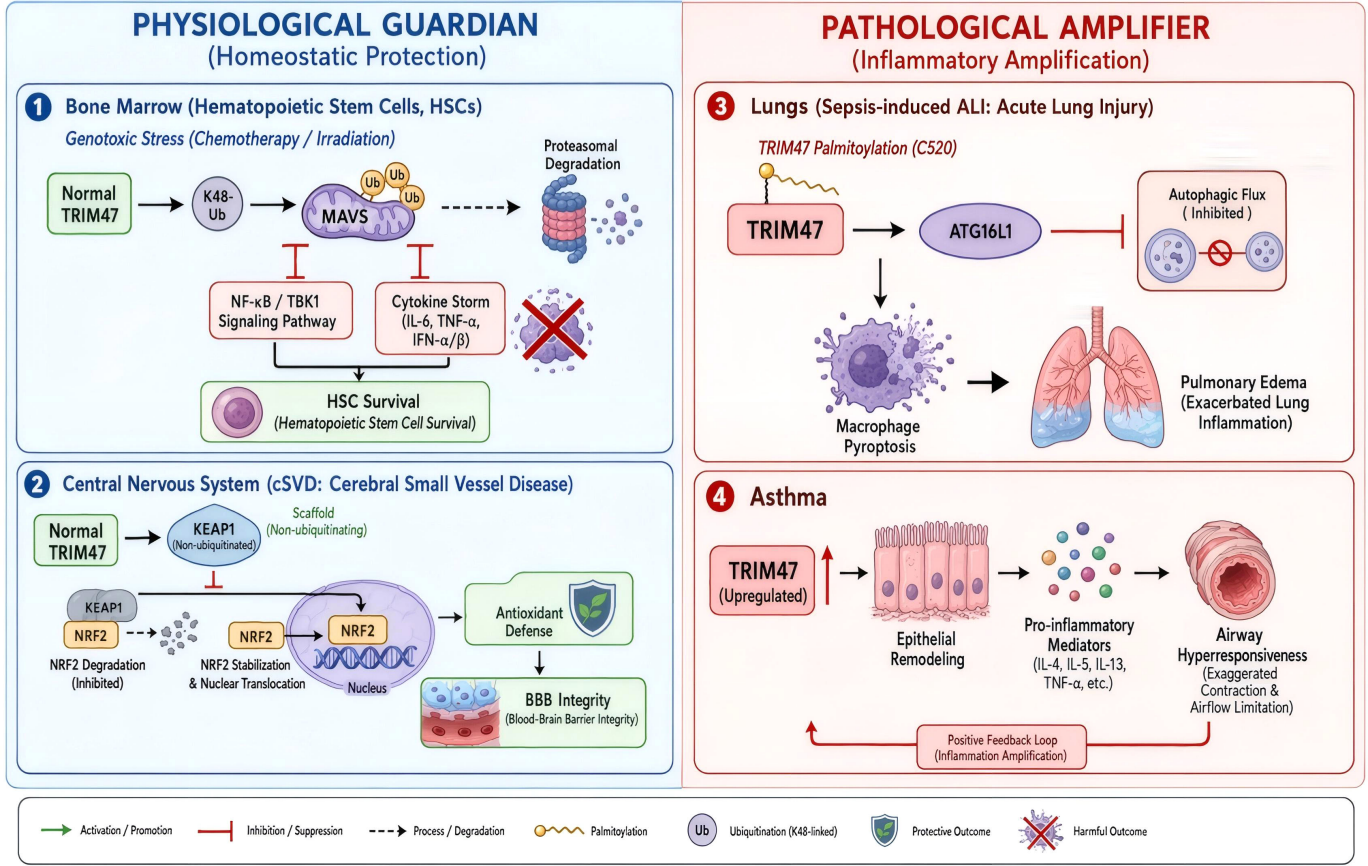
The dual role of TRIM47 in physiological homeostasis and non-neoplastic inflammatory diseases. Under steady-state or genotoxic stress, physiological TRIM47 acts as a vital “safety valve” in the bone marrow (degrading MAVS to prevent lethal IL-6/TNF-α cytokine storms) and the central nervous system (maintaining NRF2-mediated antioxidant defenses). Conversely, under specific pathogenic contexts such as sepsis-induced ALI and asthma, altered TRIM47 transforms into an “inflammatory amplifier” by blocking autophagic flux and orchestrating hyperactive inflammatory responses.

Beyond the bone marrow compartment, TRIM47 serves contrasting but equally critical roles in the brain and the lungs. Cerebral small vessel disease (cSVD) is a major global cause of stroke and vascular dementia, primarily driven by severe endothelial dysfunction and the subsequent breakdown of the blood-brain barrier (BBB) (39). In brain microvascular endothelial cells, TRIM47 acts as a crucial neurovascular protector (41). It directly binds to KEAP1, effectively preventing the pathological degradation of the master antioxidant transcription factor NRF2 (39). Depletion of TRIM47 disrupts this vital antioxidant defense, causing uncontrolled oxidative stress, intense reactive astrogliosis, and massive BBB leakage, culminating in severe cognitive impairment in *in vivo* models (39). Conversely, in the respiratory system, sepsis-induced acute lung injury (ALI) and acute respiratory distress syndrome (ARDS) are characterized by overwhelming runaway inflammation and rapid alveolar damage (54). In stark contrast to its immunosuppressive role in the bone marrow, TRIM47 functions as an inflammatory amplifier in the lungs during severe sepsis (46, 47). Triggered by ZDHHC21-mediated palmitoylation at the C520 site, TRIM47 alters its cellular localization to ubiquitinate and inhibit the autophagy-related protein ATG16L1. This effectively blocks autophagic flux, thereby accelerating macrophage pyroptosis and significantly exacerbating pulmonary edema and systemic shock (46, 47). Furthermore, asthma is a highly prevalent chronic respiratory disease characterized by airway hyperresponsiveness and structural airway remodeling heavily driven by immune dysregulation (93–95). In asthmatic models, upregulated TRIM47 orchestrates the hyperactivation of localized immune responses and airway epithelial remodeling, exacerbating the continuous release of pro-inflammatory mediators and worsening lung function, thereby acting as a critical aggravator of chronic airway disease (43) (**Figure 5**).

## 4 Clinical translational potential and obstacles

### 4.1 A robust pan-cancer predictive biomarker

The ubiquitous upregulation of TRIM47 across highly diverse human malignancies establishes it as an exceptionally robust clinical biomarker (45, 96). Prognostically, its elevated expression independently correlates with aggressive clinico-pathological features—such as deep myometrial invasion, high WHO grading, and extensive lymphatic spread—predicting significantly shortened overall survival across both solid tumors and hematological malignancies (57–60). More importantly, from a predictive standpoint, high TRIM47 levels directly identify patient cohorts that are highly likely to exhibit profound resistance to standard first-line therapies. By monitoring TRIM47 expression, clinicians could potentially refine preoperative risk stratification and identify patients who might benefit from alternative or intensified treatment regimens, moving closer to the goal of personalized oncology (64–66).

### 4.2 Clinical prognostic value

The ubiquitous upregulation of TRIM47 across diverse human malignancies establishes it as an exceptionally robust pan-cancer prognostic biomarker. Multivariate survival analyses from multiple independent cohorts consistently identify high TRIM47 expression as an independent predictor of adverse clinical outcomes, providing critical evidence for its integration into precision oncology workflows.

In gastric cancer, long-term follow-up data from a multicenter cohort of 243 patients demonstrate that the high-TRIM47-expression group exhibits significantly shorter overall survival (OS) and disease-free survival (DFS) than the low-expression group (97). Multivariate Cox regression analysis further confirms that high TRIM47 expression represents an independent risk factor for poor prognosis (hazard ratio [HR]=2.356, 95% confidence interval [CI]: 1.423–3.897, *P* < 0.001). In intrahepatic cholangiocarcinoma, transcriptomic profiling reveals that TRIM47 expression is significantly elevated (log fold change=2.473, *p* < 0.001), with high expression associated with shorter disease-free survival and serving as an independent poor prognostic factor (92).

In breast cancer, a retrospective analysis of 116 patients receiving tamoxifen endocrine therapy reveals significantly higher recurrence rates in the high-TRIM47-expression group (*P* = 0.012), indicating its utility in predicting not only overall prognosis but also specific response to endocrine therapy (13). Ovarian cancer clinical studies similarly demonstrate that high TRIM47 expression correlates significantly with poor prognosis, with high-expression patients exhibiting markedly shortened progression-free survival; multivariate analysis confirms its status as an independent prognostic predictor (HR = 2.63) (7, 98).

In glioma, survival analysis leveraging The Cancer Genome Atlas (TCGA) and Chinese Glioma Genome Atlas (CGGA) databases consistently shows significantly shortened overall survival in high-TRIM47-expression patients, with stable prognostic predictive value across different WHO grades (99). Additionally, clinical data from gallbladder cancer, osteosarcoma, and intrahepatic cholangiocarcinoma independently confirm that elevated TRIM47 expression serves as a robust indicator for predicting poor survival outcomes (45, 64, 92).

These convergent findings establish TRIM47 as possessing dual clinical value: first, as a pan-cancer independent prognostic factor providing critical references for prognosis assessment and treatment decision-making; and second, as a diagnostic-related marker with potential for integration with liquid biopsy techniques (e.g., circulating tumor DNA, exosomal RNA) and radiomics features to construct multi-modal predictive models for early tumor screening, therapeutic monitoring, and recurrence surveillance.

### 4.3 Precision nanomedicine: the siTRIM47@PD delivery system

Given the undeniable oncogenic and chemo-resistant role of TRIM47, its therapeutic silencing within the tumor microenvironment is highly desirable. However, conventional gene therapy has long been hindered by extremely low delivery efficiency and rapid *in vivo* clearance. A recent major breakthrough addressing these translational hurdles is the development of the siTRIM47@PD nanoparticle system. The siTRIM47@PD system is a sophisticated, biodegradable polymeric nanocarrier (measuring approximately 80–120 nm in diameter) specifically designed to encapsulate TRIM47 small interfering RNA (siRNA) (11). It ingeniously achieves highly efficient dual-targeting: it passively accumulates in solid tumor tissues by exploiting the Enhanced Permeability and Retention (EPR) effect, and it actively binds to hepatocellular carcinoma cells through specific surface ligands targeting the Asialoglycoprotein Receptor (ASGPR), which is uniquely and highly overexpressed on hepatocyte membranes (9). Upon entering the acidic endosomal microenvironment of the targeted tumor cell, the nanoparticle undergoes responsive structural degradation, rapidly releasing the siRNA to execute highly efficient silencing of intracellular TRIM47 expression (16). Notably, *in vivo* preclinical studies definitively demonstrate that siTRIM47@PD profoundly inhibits liver tumor growth and systemic metastasis. Crucially, unlike the catastrophic systemic effects observed in whole-body Trim47 knockout mice, animals treated with siTRIM47@PD exhibited absolutely no significant off-target toxicity. There was no observed body weight loss, no histopathological damage to major organs, no elevation of liver damage enzymes (ALT/AST), and most importantly, no systemic elevation of inflammatory cytokines (11). This demonstrates a highly favorable biosafety profile, proving that tumor-specific targeted delivery of TRIM47 inhibitors is both conceptually feasible and biologically safe.

### 4.4 Therapeutic strategies targeting TRIM47

The druggability of TRIM47 is supported by its pan-cancer expression pattern, its clear gain-of-function oncogenic phenotype, and the validation that genetic knockdown profoundly inhibits tumor growth across multiple preclinical models (11, 45, 57, 66). Translational strategies targeting TRIM47 can be categorized into three complementary approaches: direct enzymatic inhibition, protein degradation technologies, and combination regimens that exploit synthetic lethality.

#### 4.4.1 Development of small-molecule inhibitors

Small-molecule inhibitors represent an important direction for targeting TRIM47, encompassing two mechanistic categories. The first category targets the catalytic RING domain to block its interaction with E2 ubiquitin-conjugating enzymes, thereby inhibiting E3 ligase catalytic function (3). The second category comprises protein-protein interaction (PPI) blockers that disrupt specific TRIM47-substrate binding interfaces, offering higher selectivity by sparing physiological TRIM47-MAVS interactions. In hepatocellular carcinoma, the small-molecule compound CZ-2401 specifically interferes with the TRIM47-HNF4α interaction, inhibiting HNF4α K48-linked ubiquitination and degradation, stabilizing HNF4α protein, and suppressing tumor progression (100). This compound has entered preclinical research, providing critical proof-of-concept for TRIM47-specific inhibitor development. Given that TRIM47 can ubiquitinate and regulate the stability of various tumor suppressor proteins—including PPM1A, CYLD, and FBP1—to drive tumorigenesis and drug resistance, targeting its E3 ligase activity or substrate binding interface possesses broad antitumor therapeutic value (7, 101).

#### 4.4.2 PROTAC technology application

Given that TRIM47 possesses both E3 ubiquitin ligase activity and signaling scaffold functions, simple enzymatic inhibition may be insufficient to completely block its oncogenic effects. PROTAC (proteolysis-targeting chimeras) technology can induce TRIM47’s own ubiquitination and degradation through bifunctional molecules, simultaneously eliminating both enzymatic and scaffold functions (60, 102). Although no PROTACs directly targeting TRIM47 have been reported, structural studies of other TRIM family members—such as the C-degron-based ligand development for TRIM7—can provide critical technical references for their design (103). Multi-cancer studies indicate that TRIM47 represents a potential target for both small-molecule inhibitors and PROTACs (66, 67), and TRIM47 knockdown in animal models significantly inhibits tumor growth, confirming its druggability (45). Future research must focus on screening and optimization of specific PROTAC molecules, linker design, and pharmacokinetic assessment to accelerate clinical translation.

#### 4.4.3 Combination therapeutic strategy exploration

Combining TRIM47 inhibitors with existing standard therapies represents a primary direction for clinical translation, achieving synergistic efficacy through multi-target, multi-mechanism approaches. Such combinations include: (i) combination with chemotherapy to enhance chemosensitivity by reversing metabolic reprogramming (e.g., FBP1 degradation-mediated Warburg effect) and inhibiting DNA damage repair (e.g., PARP1 stabilization) (15, 63); (ii) combination with immune checkpoint inhibitors to reverse immunosuppressive microenvironments, particularly the M2-type macrophage polarization driven by TRIM47-mediated metabolic enzyme degradation (37); and (iii) combination with targeted drugs to block bypass signaling and delay targeted therapy resistance, exemplified by the PKCϵ/PKD3-NF-κB axis in endocrine-resistant breast cancer (13). These combination strategies are conceptually aligned with evolutionary trap theory, wherein targeting TRIM47 may lure cancer cells into vulnerable phenotypic states that are subsequently eradicated by standard therapies (104).

### 4.5 The therapeutic dilemma: systemic toxicity vs. tumor selectivity

The stark biological contrast between the lethal phenotype of systemic Trim47 knockout and the remarkable safety of the targeted siTRIM47@PD nanomedicine underscores the central dilemma in targeting TRIM47: the selectivity-toxicity paradox (11). The development of systemic, small-molecule inhibitors targeting the catalytic RING domain of TRIM47 remains extraordinarily risky for human translation. Because physiological TRIM47 is the master safety valve preventing MAVS-driven, IL-6/TNF-α-mediated lethal cytokine storms in the bone marrow, and the primary protector of NRF2 at the blood-brain barrier, unselective systemic inhibition could rapidly trigger lethal bone marrow failure and catastrophic neurotoxicity in patients (29, 39). Furthermore, the high structural homology of the catalytic RING domain among the 70+ members of the human TRIM family makes avoiding off-target cross-reactivity a daunting biochemical challenge (8). Therefore, to safely translate TRIM47 targeting into clinical practice, future therapeutic research must strictly pivot away from systemic ligase inhibitors. The field must prioritize expanding advanced microenvironment-responsive delivery vehicles, such as siTRIM47@PD, to confine inhibition strictly within the TME. Additionally, developing Protein-Protein Interaction (PPI) blockers (e.g., small molecules like CZ-2401) that selectively block specific pathological substrate-binding interfaces without disrupting the TRIM47-MAVS physiological interaction represents a highly promising future direction (**Table 3**).

**Table 3:** Therapeutic strategies targeting TRIM47.

Strategy category	Specific approach	Target/mechanism	Development stage	TRIM47-specific pros and cons	References
Direct enzymatic inhibition	Small molecules targeting RING domain	Blocks E2–RING interaction to inhibit ligase activity	Hit identification	Pros: Direct inhibition; Cons: High off-target/toxicity risk	(3)
Protein–protein interaction blockade	PRY-SPRY or LXXLL inhibitors	Blocks substrate binding (e.g., HNF4α, FBP1)	Preclinical	Pros: High specificity; Cons: Interface difficult to target	(3, 10)
Protein degradation	PROTAC/molecular glues	Induces TRIM47 degradation via VHL/CRBN	Proof-of-concept	Pros: Complete elimination; Cons: Poor CNS penetration	(11)
Upstream intervention	CARM1 inhibitors; ZDHHC21 inhibitors	Blocks PTMs to restore TRIM47 degradation	Preclinical	Pros: Physiological regulation; Cons: Broad substrate spectrum	(21, 25)
Combination strategy	TRIM47 inhibition + immunotherapy	Reverses M2 immunosuppression; promotes CD8+ T-cell infiltration	Preclinical	Pros: Enhances efficacy; Cons: Autoimmune risk	(38)
	TRIM47 inhibition + chemotherapy/radiotherapy	Sensitizes therapy by inhibiting DNA repair and metabolism	Preclinical	Pros: Reduces dose; Cons: Requires timing optimization	(15, 64)

TRIM47, tripartite motif-containing 47; PTMs, post-translational modifications; PROTAC, proteolysis-targeting chimera; PPI, protein–protein interaction; EMT, epithelial–mesenchymal transition; mCRPC, metastatic castration-resistant prostate cancer; ER+, estrogen receptor-positive; ATC, anaplastic thyroid cancer; HCC, hepatocellular carcinoma; ICC, intrahepatic cholangiocarcinoma; PDAC, pancreatic ductal adenocarcinoma; HNSCC, head and neck squamous cell carcinoma; ALI, acute lung injury; NF-κB, nuclear factor κB; TGF-β, transforming growth factor-β.

### 4.6 Challenges in clinical translation and potential mitigation strategies

Despite the theoretical attractiveness of targeting TRIM47, its clinical translation path is fraught with substantial challenges, and current discussions remain largely at the preclinical and conceptual level. First, drug discovery faces severe technical bottlenecks. The RING domain of TRIM47 is highly conserved across the 70+ members of the human TRIM family, presenting substantial difficulties for developing highly selective, potent small-molecule inhibitors that avoid off-target cross-reactivity (3, 14). Although PROTAC and other degradation technologies offer new approaches to eliminate TRIM47’s multiple functions—including its scaffold functions—their design, optimization, and potential “off-target” degradation risks have not been fully assessed, and no relevant molecules have entered preclinical development (103).

Second, and more critically, potential on-target toxicity risks cannot be ignored. As elaborated in preceding sections, physiological TRIM47 is the master safety valve preventing MAVS-driven, IL-6/TNF-α-mediated lethal cytokine storms in the bone marrow, and the primary protector of NRF2-mediated antioxidant defenses at the blood-brain barrier (29, 41). Therefore, systemic, non-selective inhibition or degradation of TRIM47 could theoretically lead to severe adverse effects including bone marrow suppression, immunodeficiency, and catastrophic neurotoxicity (100). The “therapeutic window” must serve as a core evaluation metric when considering any targeting strategy. The stark biological contrast between the lethal phenotype of whole-body Trim47 knockout mice and the remarkable safety of tumor-targeted siTRIM47@PD nanomedicine underscores that the selectivity-toxicity paradox is the central dilemma in TRIM47 drug development (11).

To safely translate TRIM47 targeting into clinical practice, future research must strictly pivot away from systemic ligase inhibitors and prioritize two mitigation strategies. The first is the expansion of advanced tumor microenvironment-responsive delivery vehicles—such as the siTRIM47@PD nanoparticle system—to confine inhibition strictly within the tumor microenvironment, thereby sparing physiological TRIM47 functions in HSCs and brain endothelial cells (11). The second is the development of Protein-Protein Interaction (PPI) blockers (e.g., small molecules like CZ-2401) that selectively block specific pathological substrate-binding interfaces without disrupting the TRIM47-MAVS physiological interaction (100). Additionally, thorough exploration of TRIM47 “non-oncogene addiction” functions in different cell types, combined with specific biomarkers (e.g., CARM1 methylation status, co-expression profiles), is prerequisite for achieving precise patient stratification and ensuring that therapeutic benefits outweigh risks (9, 21).

## 5 Summary

TRIM47 is an E3 ubiquitin ligase characterized by RING, B-box, coiled-coil, and B30.2/SPRY domains, enabling flexible catalysis of both K48-linked and K63-linked ubiquitination. Under physiological conditions, TRIM47 maintains immune and neurovascular homeostasis by degrading MAVS in hematopoietic stem cells and stabilizing NRF2-mediated antioxidant defenses in brain endothelial cells. In the tumor microenvironment, post-translational modifications and altered substrate availability reprogram TRIM47 toward the degradation of tumor suppressors and metabolic enzymes, while persistently activating NF-κB and Wnt/β-catenin signaling. This functional transformation drives malignant progression, metabolic reprogramming, multidrug resistance, and immunosuppressive microenvironment remodeling across diverse malignancies. Clinically, elevated TRIM47 expression correlates with advanced tumor stage, metastasis, and poor prognosis, establishing its potential as a pan-cancer biomarker. However, the high conservation of its RING domain and its indispensable physiological roles in hematopoietic and neurovascular homeostasis pose substantial challenges for systemic therapeutic targeting.

## 6 Future perspectives

Several critical gaps must be addressed to advance the TRIM47 field from mechanistic description to clinical application. First, high-resolution structural studies of TRIM47-E2-substrate complexes are still lacking; resolving these structures is essential to decode the molecular logic of linkage selection and to enable structure-based drug design. Second, tissue-specific functional profiles of TRIM47 across tumor cells, immune cells, and stem cells remain undefined; constructing conditional knockout models combined with multi-omics approaches will be necessary to map these profiles and to delineate the therapeutic window Third, the structural conservation of the RING domain across the TRIM family continues to impede the development of selective catalytic inhibitors; future efforts should prioritize allosteric inhibitors and tumor-targeted PROTACs to circumvent off-target toxicity. Fourth, prospective clinical validation of TRIM47 as a prognostic biomarker and therapeutic target remains limited to retrospective cohorts; large-scale, multi-center prospective trials are urgently needed. Finally, given the immunotherapy focus of this special issue, dissecting the bidirectional crosstalk between TRIM47-driven metabolic reprogramming and immune checkpoint blockade efficacy represents a particularly promising frontier. Elucidating whether TRIM47 inhibition can reverse M2 macrophage polarization and convert “cold” tumors into “hot” tumors may unlock synergistic combination strategies that expand the immunotherapy responder population.

## 7 Conclusion

TRIM47 is not merely a canonical E3 ubiquitin ligase; it is a master contextual switch governed by cellular environments and post-translational modifications. In normal physiology, it acts as a steadfast guardian of immune and neurovascular homeostasis, preventing stress-induced cytokine storms and maintaining barrier integrity. In malignancy, hijacked by altered microenvironments, it transforms into an aggressive driver of metabolic reprogramming, immune evasion, and multi-drug resistance across diverse organ systems. While its pan-cancer biomarker value is ready for large-scale clinical validation, unlocking its direct therapeutic potential demands exquisite precision. Only by leveraging advanced tumor-targeted nanomedicine and selective protein-protein interaction inhibitors can we safely exploit the oncogenic vulnerabilities of TRIM47 without unleashing the devastating inflammatory consequences of its physiological depletion. Continued investigation of this pivotal molecule will not only deepen our understanding of E3 ligase functional plasticity but also provide novel strategies for precision diagnosis and treatment of TRIM47-dependent malignancies.

## Glossary

5-FU: 5-Fluorouracil
ADAR: Adenosine Deaminase Acting on RNA
AKT: Protein Kinase B
ALI: Acute Lung Injury
ARDS: Acute Respiratory Distress Syndrome
AR: Androgen Receptor
ASGPR: Asialoglycoprotein Receptor
ATC: Anaplastic Thyroid Cancer
ATF6: Activating Transcription Factor 6
ATG16L1: Autophagy Related 16 Like 1
BBB: Blood-Brain Barrier
BC: Breast Cancer
CARM1: Coactivator-Associated Arginine Methyltransferase 1
CDO1: Cysteine Dioxygenase 1
CGGA: Chinese Glioma Genome Atlas
CRL4: Cullin 4-RING E3 ligase
CRPC: Castration-Resistant Prostate Cancer
cSVD: Cerebral Small Vessel Disease
CYLD: CYLD Lysine 63 Deubiquitinase
DFS: Disease-Free Survival
DTC: Differentiated Thyroid Cancer
EMT: Epithelial-Mesenchymal Transition
ER: Estrogen Receptor
EPR: Enhanced Permeability and Retention
FBP1: Fructose-1,6-Bisphosphatase 1
FH: Fumarate Hydratase
GBC: Gallbladder Cancer
GC: Gastric Cancer
GBM: Glioblastoma
HCC: Hepatocellular Carcinoma
HIF: Hypoxia-Inducible Factor
HNSCC: Head and Neck Squamous Cell Carcinoma
HPV: Human Papillomavirus
HSC: Hematopoietic Stem Cell
ICC: Intrahepatic Cholangiocarcinoma
ICAM-1: Intercellular Adhesion Molecule-1
IFN-I: Type I Interferon
IL-1β: Interleukin-1 beta
IL-6: Interleukin-6
KEAP1: Kelch-Like ECH-Associated Protein 1
LPS: Lipopolysaccharide
MAVS: Mitochondrial Antiviral Signaling Protein
mCRPC: Metastatic Castration-Resistant Prostate Cancer
METTL3: Methyltransferase-Like 3
MDM2: Mouse Double Minute 2 Homolog
NEMO: NF-κB Essential Modulator
NF-κB: Nuclear Factor Kappa B
NLRP3: NLR Family Pyrin Domain Containing 3
NSCLC: Non-Small Cell Lung Cancer
NRF2: Nuclear Factor Erythroid 2-Related Factor 2
OC: Ovarian Cancer
OS: Overall Survival/Osteosarcoma
PARP1: Poly (ADP-Ribose) Polymerase 1
PCa: Prostate Cancer
PKC-ϵ: Protein Kinase C Epsilon
PKD3: Protein Kinase D3
PPM1A: Protein Phosphatase Mg2+/Mn2+ Dependent 1A
PROTAC: Proteolysis Targeting Chimera
PRY-SPRY: PRY-SPRY Domain (B30.2 domain)
PTMs: Post-translational Modifications
RAI: Radioactive Iodine
RING: Really Interesting New Gene
siRNA: Small Interfering RNA
SNAI1: Snail Family Transcriptional Repressor 1
SOFA: Sequential Organ Failure Assessment
STAT3: Signal Transducer and Activator of Transcription 3
TCGA: The Cancer Genome Atlas
TGF-β: Transforming Growth Factor-beta
TNF-α: Tumor Necrosis Factor Alpha
TRAF2: TNF Receptor Associated Factor 2
TRIM47: Tripartite Motif Containing 47
VCAM-1: Vascular Cell Adhesion Molecule-1
VEGF: Vascular Endothelial Growth Factor
WMH: White Matter Hyperintensities
XAF1: XIAP Associated Factor 1
ZDHHC21: Zinc Finger DHHC-Type Palmitoyltransferase 21.
